# Health Tract, No. 245

**Published:** 1866-06

**Authors:** 


HEALTH TRACT, No. 245.

### SYMPTOMS.

I suppose that in the course of my medical career I have received, literally, thousands of letters similar to the following, which came to hand April 12th, 1866, from a gentleman of position, of a superior education, and of high culture. "On account of business pertaining to my profession, I have been prevented from seeing you for several months. I am happy to inform you that I am improving; I have felt better for the last three months than I have for two years. I cannot be thankful enough for the instructions received from you. I have been busy, very busy, all this winter. Can stand the cold nearly as well as ever. I have not taken a particle of medicine since last December, except what you gave me (half a dozen pills, Ed.) My throat is nearly well. I must again thank you for your treatment. You taught me how to live, which I never knew before."

It may be instructive to make some comments on this case; this gentleman had made application six months before, had been heard from once, and not seen at all. He complained of

1. Burning and dryness in the throat.
2. On first rising in the morning his head was dull, with running from the nose and dizziness.
3. Coughing for two hours after breakfast.
4. Shifting pains in the body.
5. Raw sensation in the stomach.
6. Continued desire to eat.
7. Pain on the right side.
8. Pains back of the neck, extending to the head; when out of doors the wind seems to concentrate there.
9. Constipation.
10. Bilious.
11. Headache.
12. Belching.
13. Pains in breast.

The written opinion (always given) in this case was, "You have liver complaint, constipation and dyspeptia, reacting on one another, and you can get well, because your lungs are perfectly sound. There is no reason to doubt of your regaining your health, and living many years."

The first important step in leading to this gentleman's restoration was relieving the mind of those depressing forebodings of a dreaded disease, by showing him that it could not exist. The second was not only in showing him the impolicy of abandoning his profession even temporarily, but that it was important for him to follow it with a new energy, to have his mind fully occupied with it, even to be a little driven. It is almost impossible for an active cultivated mind to get well of any serious ailment, if the patient is placed in a condition which allows him to lounge, and loll and mope about, hanging about the house, the mind all the time reverting to the bodily ailments, going round and round in the same track, as in a horse-mill.

Third. The mode of a man's life as to eating, sleeping, clothing, exercise and employment of time.

Fourth. A pill or two a month to relieve the system of what clogged the working of the machinery until it could get a fair start, and then to rely on general hygienic rules of life.

